# Agreement of cerebrospinal fluid biomarkers and amyloid-PET in a multicenter study

**DOI:** 10.1007/s00406-023-01701-y

**Published:** 2023-10-28

**Authors:** Núria Guillén, José Contador, Mariateresa Buongiorno, Ignacio Álvarez, Natalia Culell, Daniel Alcolea, Alberto Lleó, Juan Fortea, Gerard Piñol-Ripoll, Anna Carnes-Vendrell, María Lourdes Ispierto, Dolores Vilas, Albert Puig-Pijoan, Aida Fernández-Lebrero, Mircea Balasa, Raquel Sánchez-Valle, Albert Lladó

**Affiliations:** 1 Alzheimer’s Disease and Other Cognitive Disorders Unit, Institut d’Investigacions Biomèdiques August Pi i Sunyer (IDIBAPS), Hospital Clínic de Barcelona, Carrer Villarroel, 170, 08036 Barcelona, Spain; 2 Memory Disorders Unit, Department of Neurology, Hospital Universitari Mutua de Terrassa, Terrassa, Spain; 3 Fundació Docència i Recerca Mútua Terrassa, Terrassa, Spain; 4 Sant Pau Memory Unit, Department of Neurology, Hospital de la Santa Creu i Sant Pau-Biomedical Research Institute Sant Pau (IIB Sant Pau), Universitat Autònoma de Barcelona, Barcelona, Spain; 5 Centro de Investigación Biomédica en Red de Enfermedades Neurodegenerativas. CIBERNED, Madrid, Spain; 6 Clinical Neuroscience Research, Unitat Trastorns Cognitius, IRBLleida, Santa Maria University Hospital, Lleida, Spain; 7 Neurodegenerative Diseases Unit, Neurology Service and Neurosciences Department, University Hospital Germans Trias i Pujol (HUGTP), Badalona, Spain; 8 Cognitive Decline and Movement Disorders Unit, Neurology Department, Hospital del Mar, Barcelona, Spain; 9 Integrative Pharmacology and Systems Neurosciences Research Group, Neurosciences Research Program, Hospital del Mar Medical Research Institute (IMIM), Barcelona, Spain; 10 Institute of Neurosciences, Department of Medicine, Faculty of Medicine and Health Sciences, University of Barcelona, Barcelona, Spain

**Keywords:** Alzheimer’s disease, Biomarkers, Amyloid-PET, CSF

## Abstract

Core Alzheimer’s disease (AD) cerebrospinal fluid (CSF) biomarkers have shown incomplete agreement with amyloid-positron emission tomography (PET). Our goal was to analyze the agreement between AD CSF biomarkers and amyloid-PET in a multicenter study. Retrospective multicenter study (5 centers). Participants who underwent both CSF biomarkers and amyloid-PET scan within 18 months were included. Clinical diagnoses were made according to latest diagnostic criteria by the attending clinicians. CSF Amyloid Beta_1-42_ (Aβ_1-42_, A), phosphorliated tau 181 (pTau181, T) and total tau (tTau, N) biomarkers were considered normal (−) or abnormal ( +) according to cutoffs of each center. Amyloid-PET was visually classified as positive/negative. Agreement between CSF biomarkers and amyloid-PET was analyzed by overall percent agreement (OPA). 236 participants were included (mean age 67.9 years (SD 9.1), MMSE score 24.5 (SD 4.1)). Diagnoses were mild cognitive impairment or dementia due to AD (49%), Lewy body dementia (22%), frontotemporal dementia (10%) and others (19%). Mean time between tests was 5.1 months (SD 4.1). OPA between single CSF biomarkers and amyloid-PET was 74% for _Aβ1-42_, 75% for pTau181, 73% for tTau. The use of biomarker ratios improved OPA: 87% for Aβ_1-42_/Aβ_1-40_ (*n* = 155), 88% for pTau181/Aβ_1-42_ (*n* = 94) and 82% for tTau/Aβ_1-42_ (*n* = 160). A + T + N + cases showed the highest agreement between CSF biomarkers and amyloid-PET (96%), followed by A-T-N- cases (89%). Aβ_1-42_/Aβ_1-40_ was a better marker of cerebral amyloid deposition, as identified by amyloid tracers, than Aβ_1-42_ alone. Combined biomarkers in CSF predicted amyloid-PET result better than single biomarkers.

**Supplementary Information:**

The online version contains supplementary material available at 10.1007/s00406-023-01701-y.

## Introduction

Alzheimer’s disease (AD) is the most common cause of dementia, affecting 35 million people worldwide, with a stable or even decreasing incidence and prevalence [1] and representing a huge burden for health-care systems. In the past year, two modifying disease treatments for AD, Aducanumab and Lecanemab, have been approved by the Food and Drug Administration for the treatment of Alzheimer’s disease, and many more are being tested. To receive them, it is compulsory that AD is confirmed biologically. Autopsy series have shown that approximately 30% of AD cases are misdiagnosed [2].

Diagnostic accuracy can be improved using AD biomarkers [3], such as cerebrospinal fluid (CSF) immunoassays and amyloid positron emission tomography scan (amyloid-PET). These biomarkers are already included in the National Institute on Aging and Alzheimer’s Association (NIA-AA) diagnostic criteria 2011 and are pivotal in the biological definition of AD in the AT(N) research framework [4]. A precise diagnosis helps physicians to guide available therapy and to properly advise patients and caregivers. AD biomarkers also permit to identify subjects in preclinical stages and with mild cognitive impairment at risk of evolution to AD dementia, which could benefit from a disease-modifying therapy before the effects of neurodegeneration are established.

Under the biological definition of AD in the AT(N) research framework, biomarkers are grouped into those of β amyloid deposition (A), pathologic tau (T), or neurodegeneration (N). The “A” category is defined by amyloid Beta_1-42_ (Aβ_1-42_) CSF levels, Aβ_1-42_/Aβ_1-40_ ratio in CSF or amyloid-PET, “T” by phosphorylated tau (pTau) CSF levels or tau-PET and “N” by total tau (tTau) CSF levels, structural cranial magnetic resonance (MRI) or 18F-fluorodeoxyglucose-PET. In clinical practice, to obtain a diagnosis of AD with high certainty, the available amyloid biomarkers are CSF amyloid biomarkers and amyloid-PET. Both are surrogate markers of the presence of amyloid plaques in the brain, but in addition CSF biomarkers might provide information about the “T” and “N” categories. According to the NIA-AA algorithm, each biomarker value can be classified as positive for AD, negative or borderline [4].

CSF biomarkers have shown good, but not complete, concordance with amyloid-PET classification [5–7]. Several causes for this discordance have been described. First, amyloid-PET and CSF analysis measure different species of Aβ: amyloid-PET ligands bind to aggregated forms of Aβ, whereas CSF immunoassays measure mainly soluble Aβ [8]. Secondly, both CSF biomarker determination and amyloid-PET scans are subject to errors and variability. There are preanalytical factors related to collection and storage procedures, and analytical factors, such as different analysis protocols and techniques, that lead to variability in the concentrations of CSF biomarkers [9–11]. Amyloid-PET results can vary due to radiotracer characteristics, subject movement, or amyloid threshold selection, among others [12], and visual evaluations are subject to rater-to-rater variability [13]. Furthermore, every center has its own protocol and cutoffs to determine positivity of AD CSF biomarkers and amyloid-PET, which could lead to differences between centers.

The general aim of this study is to analyze the agreement of AD CSF biomarkers and amyloid-PET scan results, the two available tests to detect *in-vivo* amyloid deposition in the brain, and, therefore, that allow confirming biological ADto a person with cognitive impairment. To expand the knowledge about these tests and their limitations in the diagnosis of AD, we also aimed to analyze cases in which CSF amyloid and amyloid-PET scan have results of opposite sign.

The novelty of this work lies in the fact that the concordance between CSF and Amyloid PET has been analyzed in a heterogeneous sample from 5 centers, and that the sample is enriched with difficult diagnosis cases that made the clinicians request a second amyloid test. For these reasons, we believe that the results obtained are of great interest and can be extrapolated to different scenarios.

## Methods

### Study participants

This is a retrospective multicenter study. Six centers in Catalonia, Spain (Hospital de la Santa Creu i Sant Pau, Hospital del Mar, Hospital Clínic de Barcelona, Hospital Mútua de Terrassa, Hospital Santa Maria de Lleida and Hospital Germans Trias i Pujol) were invited to participate in the study and to send data from participants studied previously. The inclusion criteria were consecutive participants studied in a dementia unit, who underwent both a lumbar puncture to analyze AD CSF biomarkers and an amyloid-PET scan between January 2016 and December 2020, with a maximum time of 18 months between both.

Demographic and clinical data were registered: age at first visit, sex, diagnosis, Mini Mental State Examination (MMSE) score, Apolipoprotein E ε4 (APOE ε4) allele status, and the reason why both tests were performed. Diagnoses were made by the attending clinicians according to the latest diagnostic criteria and considering clinical features and CSF and amyloid PET results. Participants enrolled in the study were diagnosed with mild cognitive impairment (MCI) due to AD [14], dementia due to AD [15], frontotemporal dementia (FTD) [16, 17], dementia with Lewy bodies (LBD) [18], MCI non-AD (participants with MCI not fulfilling diagnostic criteria of AD or any other neurodegenerative disorder at the time of assessment), vascular dementia[19], depression [20] and subjective cognitive decline (SCD) [21]. Five of the participants were cognitively unimpaired individuals of the Sant Pau Initiative on Neurodegeneration cohort that volunteered for investigation projects.

To analyze the performance of Elisa Innotest® and fully automated Lumipulse® we compared the percentage of correctly classified CSF biomarkers (according to amyloid-PET) in each group and performed a Fisher’s exact test.

### CSF biomarkers

CSF biomarkers were analyzed in each center according to their own protocols following manufacturer’s instructions. All analyses were performed by experienced laboratory personnel blinded to clinical diagnosis.

Requested data included Aβ_1-42_, Aβ_1-40_, pTau and tTau levels. Dates of LP and technique used for CSF analysis were registered. Each center provided their own cutoffs for CSF single biomarkers and ratios. Aβ_1-42_/Aβ_1-40,_ pTau/Aβ_1-42_ and tTau/Aβ_1-40_ ratios were analyzed only if the center had established a specific cutoff for them [22, 23]. CSF biomarkers levels were dichotomized in abnormal ( +) or normal (-) according to the cutoff points of each center. Then, we obtained the ATN profiles: A + T + N + , A-T-N-, A + T + N-, A + T–N + , A-T-N + or A-T + N-.

We also performed an exploratory analysis using a trichotomization strategy: the values within 20% of the standard deviation from the respective cutoff of each biomarker for each center were classified as borderline [24]. We analyzed the new ATN profiles resulting from trichotomization of Aβ1-42 and Aβ1-42/Aβ1-40. Results of borderline Aβ1-42 and borderline Aβ1-42/Aβ1-40 did not differ from positive Aβ1-42 and Aβ1-42/Aβ1-40.

The “A” category was assessed using Aβ_1-42_ levels and Aβ_1-42_/Aβ_1-40_ ratio when available, and both results were included in the analysis.

### Amyloid-PET

Amyloid-PET scans were performed in each center according to their own protocols and classified in positive or negative based on visual interpretation. Dates of amyloid-PET acquisition and used tracer were also registered. The choice of PET tracers was based on availability at each center. Amyloid-PET was evaluated according to current guidelines and experienced nuclear medicine physicians.

### Statistical analysis

We compared clinical and demographic characteristics of all participants based on visual amyloid-PET status using Welch two-sample t-test or Fisher’s exact test. To analyze the performance of CSF biomarkers and ratios compared to amyloid-PET visual status we performed receiver operating characteristic (ROC) analysis to calculate areas under the curve with 95% confidence interval. We determined positive percent agreement (PPA) (or sensitivity), negative percent agreement (NPA) (or specificity) and overall percent agreement (OPA) between CSF biomarker results and amyloid-PET visual interpretation. To analyze the performance of Elisa Innotest® and fully automated Lumipulse® we compared the percentage of correctly classified CSF biomarkers (according to amyloid-PET) in each group and performed a Fisher’s exact test. Data was analyzed using Stata software, Stata/IC 16.

## Results

### Demographic, biomarker, APOE and clinical data

We received data about 305 participants. Sixty-nine participants were excluded: 30 for having a time between LP and PET > 18 months, 30 for not having information about pTau and/or tTau, 7 for having a doubtful amyloid PET result and 2 for not having a final clinical diagnosis. Finally, 236 participants from 5 centers were included in the study.

Table 1 summarizes demographic, APOE and clinical data according to amyloid-PET status. Comparing amyloid-PET + and amyloid-PET- participants, there were no differences in age at first visit, sex, time difference between LP and amyloid-PET acquisition, technique used to CSF analysis, tracer used to amyloid-PET or reason for doing both a LP and an amyloid-PET scan. MMSE scores were lower in the amyloid-PET + group. As expected, there was a higher proportion of APOEε4 carriers in the amyloid-PET + group compared to the amyloid-PET- (54% and 24%, respectively). Reasons for doing both LP and amyloid-PET were: participation in an observational research project (*n* = 124, 53%) or a clinical trial (*n* = 26, 11%) which required both tests or the test not done previously or an inconclusive CSF result / unclear clinical diagnose after the first test results (*n* = 23, 10%); there were no differences between amyloid-PET + and amyloid-PET- groups. In 5 out of 23 inconclusive CSF result/unclear clinical diagnose cases the clinician was not confident with the diagnosis after the first test results and a second test was performed. In the other 18 cases, the CSF result was not definite to confirm or rule out AD and an amyloid-PET scan was performed. The most frequent profile in these cases was A + T-N- (*n* = 14), including 6 cases in which the amyloid-PET scan was negative (2 cases diagnosed as FTD, 3 as MCI non degenerative and one as MCI due to AD) and 8 cases in which the amyloid-PET scan was positive (all of them diagnosed of MCI/dementia due to AD). Other AT(N) profiles in this group were A-T + N + (*n* = 3) and A + T-N + (*n* = 1), and all had a positive amyloid-PET scan result and final diagnoses of MCI/dementia due to AD.

**Table 1:** Demographic and clinical characteristics and biomarker results, according to amyloid-PET status

	All participants	Amyloid positive	Amyloid negative	*P* value
*n* (%)	256	154 (60%)	102 (40%)	–
Age at diagnosis, years	67.6 (9.0)	68.1 (9.1)	66.9 (8.8)	0.290†
Sex, female/male (% female)	144/112 (56%)	84/70 (55%)	60/42(59%)	0.522‡
APOEε4 ± (% +) *n* = 214	88/126(41%)	67/58 (54%)	21/68 (24%)	< 0.001‡
MMSE score	24.6 (4.3)	23.9 (4.3)	25.6 (4.1)	0.003†
Time difference between amyloid-PET and LP, months	4.9 (4.0)	4.8 (4.0)	4.9.0 (4.2)	0.808†
Clinical diagnosis, *n* (%)				< 0.001‡
MCI due to AD	78 (100%)	71 (91%)	7 (9%)	–
Dementia due to AD	48 (100%)	46 (96%)	2 (4%)	–
Dementia with Lewy bodies	53 (100%)	27 (51%)	26 (49%)	–
Frontotemporal dementia	25 (100%)	1 (4%)	24 (96%)	–
MCI non degenerative	16 (100%)	0 (0%)	16 (100%)	–
MCI not specified	17 (100%)	4 (24%)	13 (76%)	
Vascular dementia	5 (100%)	1 (20%)	4 (80%)	
Depressive pseudodementia	2 (100%)	0 (0%)	2 (100%)	
Subjective memory complaints	7 (100%)	1 (14%)	6 (86%)	
Cognitively unimpaired individuals	5 (100%)	3 (60%)	2 (40%)	
CSF analysis				
Innotest®	82 (100%)	46 (56%)	36 (44%)	0.412‡
Lumipulse®	174 (100%)	108 (62%)	66 (38%)	
Reason for LP and amyloid-PET				0.334‡
Clinical assay	26 (100%)	18 (69%)	8 (31%)	–
Inconclusive CSF result/clinical diagnose unclear after the first test	27 (100%)	18 (67%)	9 (33%)	–
Investigation project	124 (100%)	73 (59%)	51 (41%)	–
Center’s own protocol	15 (100%)	12 (80%)	3(20%)	–
Not available information	64 (100%)	33 (52%)	31 (48%)	
PET tracer				0.040‡
^18^F- Florbetapir	181 (100%)	100 (55%)	81 (45%)	–
^18^F-Flutemetamol	54 (100%)	40 (74%)	14 (26%)	–
^18^F-Florbetaben	21 (100%)	14 (67%)	7 (33%)	–

Unless otherwise specified, results are presented as mean (standard deviation)

*MMSE* mini-mental state examination; *MCI* mild cognitive impairment; *AD* Alzheimer’s disease, *CSF* cerebrospinal fluid; *LP* lumbar puncture; *PET* positron emission tomography

*P*–values were calculated by comparing amyloid-positive and amyloid-negative participants using Welch two-sample *t*-test (†) or Fisher’s exact test (‡)

### Agreement between CSF biomarkers and amyloid PET

Globally, Aβ_1-42_ showed 74% OPA with amyloid-PET, pTau showed 75% OPA, and tTau showed 73% OPA. The use of a second biomarker resulted in an increase of the agreement between CSF biomarkers and amyloid PET: Aβ_1-42_/Aβ_1-40_ (*n* = 155) showed 86% OPA, pTau/Aβ_1-42_ (*n* = 94) showed 88% OPA, and tTau/Aβ_1-42_ (*n* = 160) showed 82% OPA. Global and individual center results and the areas under the curve of CSF biomarkers are presented in Table 2.

**Table 2:** Cutoffs of CSF biomarkers and agreement between CSF biomarkers and amyloid-PET

Center	CSF biomarker	Cutoff	PPA %	NPA %	OPA %	AUC (95% CI)
H. de la Santa Creu i Sant Pau (*n* = 94)	Aβ_1-42_	916 pg/mL	95	49	78	0.76 (0.65–0.86)
	pTau	63 pg/mL	80	83	81	0.83 (0.74–0.93)
	tTau	456 pg/mL	75	83	78	0.79 (0.70–0.90)
	Aβ_1-42_ /Aβ_1-40_	0,062	88	77	84	0.86 (0.77–0.96)
	pTau/Aβ_1-42_	0.068	93	80	88	0.88 (0.79–0.97)
	tTau/Aβ_1-42_	0.62	81	80	81	0.87 (0.77–0.95)
H. Mútua de Terrassa (*n* = 67)	Aβ_1-42_	629 pg/mL	49	81	64	0.80 (0.68–0.91)
	pTau	88 pg/mL	51	71	61	0.64 (0.51–0.80)
	tTau	532 pg/mL	57	69	63	0.70 (0.57–0.82)
	tTau/Aβ_1-42_	0.58	81	79	80	0.89 (0.82–0.97)
H. Clínic de Barcelona (*n* = 46)	Aβ_1-42_	600 pg/mL	96	52	78	0.84 (0.71–0.97)
	pTau	65 pg/mL	67	89	76	0.87 (0.77–0.97)
	tTau	385 pg/mL	70	84	76	0.86 (0.76–0.97)
	Aβ_1-42_ /Aβ_1-40_	0.07	100	74	89	0.95 (0.86–1.00)
H. Santa Maria de Lleida (*n* = 15)	Aβ_1-42_	600 pg/mL	82	50	73	0.81 (0.51–1.00)
	pTau	60 pg/mL	91	75	87	0.98 (0.91–1.00)
	TTau	450 pg/mL	91	100	93	0.98 (0.91–1.00)
H. del Mar (*n* = 15)	Aβ_1-42_	750 pg/mL	86	75	80	0.82 (0.58–1.06)
	pTau	69,85 pg/mL	86	75	80	0.77 (0.51–1.00)
	tTau	522 pg/mL	57	75	67	0.82 (0.60–1.00)
	Aβ_1-42_ /Aβ_1-40_	0.062	100	75	87	0.89 (0.72–1.00)
Global (*n* = 236)	Aβ_1-42_ (*n* = 236)		82	62	74	
	pTau (*n* = 236)		71	79	75	
	tTau (*n* = 236)		70	78	73	
	Aβ_1-42_ /Aβ_1-40_(*n* = 155)		92	76	86	
	pTau/Aβ_1-42_ *n* = (*n* = 94)		93	80	88	
	tTau/Aβ_1-42_ (*n* = 160)		83	80	82	

*CSF* cerebrospinal fluid; *PET* positron emission tomography; *PPA* positive percent agreement; *NPA* negative percent agreement; *OPA* overall percent agreement; *AUC* area under the curve; *Aβ*_*1-42*_ amyloid Beta_1-42_; *pTau* phosphorilated tau; *tTau* total tau

[Table 2. Cutoffs of CSF biomarkers and agreement between CSF biomarkers and amyloid-PET.]

In the subgroup of participants with available Aβ_1-42_ /Aβ_1-40_ ratio (*n* = 155), only 75% of participants with abnormal Aβ_1-42_ in CSF (*n* = 117) had a positive amyloid-PET, increasing to 87% (84/97) when the Aβ_1-42_/Aβ_1-40_ ratio was also abnormal and decreasing to 20% (4/20) when the ratio was normal. Of all participants with normal Aβ_1-42_ (*n* = 38), 87% had a negative amyloid-PET, increasing to 91% when the ratio was normal (31/34) and decreasing to 50% (2/4) when the ratio was abnormal.

### Agreement between CSF biomarkers and amyloid PET comparing ATN profiles

One hundred and twenty-three participants (52.1%) had a A + T + N + (n = 77) or A-T-N- (*n* = 46) profile and 113 (47.9%) had a A-T + /N + or A + T-/N- profile (Table 3).

**Table 3:** Agreement between CSF biomarkers and amyloid-PET

ATN status	All participants	Amyloid positive	Amyloid negative
A + T + N +	77	74 (96%)	3 (4%)
A-T-N-	46	5 (11%)	41 (89%)
A + T-N-	61	32 (52%)	29 (48%)
A + T + N-	8	5 (63%)	3 (37%)
A + T-N +	5	3 (60%)	2 (40%)
A-T + N +	31	20 (65%)	11 (35%)
A-T + N-	3	0	3 (100%)
A-T-N +	5	0	5 (100%)

*CSF* cerebrospinal fluid; *PET* positron emission tomography; *Aβ*_*1-42*_ amyloid Beta_1-42_

[Table 3. Agreement between CSF biomarkers and amyloid-PET.]

Summarized data according to ATN profile can be found in Table 1 Supplementary. In the A + T + N + group, 74/77 (96%) participants showed a positive amyloid-PET scan. A + T + N + participants showed the highest proportion of APOEε4 (52%), the lowest mean MMSE (23.5 (SD 4.5)) and AD diagnosis (MCI or dementia due to AD) represented 75% of diagnoses in this group. 17 participants in this group were diagnosed with dementia with Lewy bodies following McKeith criteria and AD was considered co-pathology. Three out of 77 participants with the A + T + N + profile presented a negative amyloid-PET scan: 60- and 73-year-old males with diagnosis of AD and a 71-year-old male with diagnosis of LBD; in the three cases Aβ_1-42_/Aβ_1-40_, pTau/Aβ_1-42_ and tTau/Aβ_1-42_ were also positive. In the A-T-N- group, 41/46 (89%) participants had a negative amyloid-PET scan. A-T-N- participants showed the lowest proportion of APOEε4 (20%), the highest mean MMSE (25.9 (SD 2.8)) and AD diagnosis only accounted for 9% of participants in this group. Five participants out of 46 with A-T-N- had a positive amyloid-PET, they had final diagnoses of MCI or dementia due to AD (*n* = 3), LBD (*n* = 1) and MCI non-AD (*n* = 1); in all cases tTau/Aβ_1-42_ (plus Aβ_1-42_/Aβ_1-40_, pTau/Aβ_1-42_ in one case) were also negative. In the A + T-/N- group, 40/74 (54%) participants had a positive amyloid-PET scan and 34 (46%) had a negative amyloid-PET scan. 46% of participants carried the APOEε4 allele, mean MMSE was 25.0 (SD 4.6) and AD diagnosis accounted for 44% of participants. In the A-T + /N + group, 20/39 (51%) participants had a positive amyloid-PET, and 19 participants (49%) had a negative amyloid-PET. 35% participants carried the APOEε4 allele, mean MMSE was 24.2 (SD 4.6) and AD diagnosis accounted for 54% of participants. The agreement between Aβ_1-42_ CSF and PET was very high in A + T + N + / A-T-N- profiles (PPA 96%, NPA 89%, OPA 93%), but low in A + T-/N- and A-T + /N + profiles (PPA 54%, NPA 49%, OPA 52%). In the subgroup of participants with available Aβ_1-42_ /Aβ_1-40_ ratio (n = 155), when Aβ_1-42_ /Aβ_1-40_ ratio was used to assess the A category instead of Aβ_1-42_ alone, 3 more participants were included in the A + T + N + group (2 of them with a positive amyloid-PET) and 19 more participants in the A-T-N- group (15 of them with a negative amyloid-PET) (Table 4). Comparing agreement of CSF biomarkers with amyloid-PET in this subgroup of participants, in A + T + N + and A-T-N- subgroups, Aβ_1-42_/Aβ_1-40_ ratio (PPA 94%, NPA 89%, OPA 92%) showed a similar performance to Aβ_1-42_ (PPA 95%, NPA 94%, OPA 95%), whereas in A + T-/N- and A-T + /N + profiles the performance of Aβ_1-42_/Aβ_1-40_ ratio (PPA 67%, NPA 78%, OPA 69%) improved compared to Aβ_1-42_ (PPA 50%, NPA 67%, OPA 53%).

**Table 4:** Agreement between CSF biomarkes and amyloid-PET

ATN status (AB _1–42_)	All participants	Amyloid positive	Amyloid negative
A + T + N +	65	62 (95%)	3 (5%)
A-T-N-	26	1 (4%)	25 (96%)
A + T-N-	42	20 (48%)	22 (52%)
A + T + N-	8	5 (63%)	3 (37%)
A + T-N +	2	1 (50%)	1 (50%)
A-T + N +	7	4 (57%)	3 (43%)
A-T + N-	1	0	1 (100%)
A-T-N +	4	0	4 (100%)
ATN status Aβ_1-42_ /Aβ_1-40_			
A + T + N +	68	64 (94%)	4 (6%)
A-T-N-	45	5 (11%)	40 (89%)
A + T-N-	23	16 (70%)	7 (30%)
A + T + N-	8	5 (63%)	3 (37%)
A + T-N +	2	1 (50%)	1 (50%)
A-T + N +	4	2 (50%)	2 (50%)
A-T + N-	1	0	1 (100%)
A-T-N +	4	0	4 (100%)

Comparison of AB_1-42_ vs Aβ_1–42_ /Aβ_1–40_

*CSF* cerebrospinal fluid; *PET* positron emission tomography; *Aβ*_*1-42*_ amyloid Beta_1-42;_ *Aβ*_*1-40*_ amyloid Beta_1-40_

[Table 4. Agreement between CSF biomarkers and amyloid-PET. Comparison of AB_1-42_ vs Aβ_1-42_ /Aβ_1-40_].

### Agreement between CSF Aβ_1-42_ with amyloid-PET by diagnosis

We analyzed the agreement between CSF Aβ_1-42_ and amyloid-PET by clinical diagnosis through OPA. Of note, reported diagnoses are after the biomarker results. In MCI due to AD, Aβ_1-42_ had OPA of 80% (95% in A + T + N + , 61% in other ATN profiles), in dementia due to AD OPA of 67% (87% in A + T + N + , 48% in other ATN profiles), in LBD OPA of 75% (94% in A + T + N + , 93% in A-T-N-, 48% in other ATN profiles), in FTD OPA of 61% (100% in A-T-N-, 44% in other ATN profiles), in MCI non-AD OPA of 70,6%, in subjective memory complaints OPA of 100%, in vascular dementia OPA of 75%, in cognitively normal controls OPA of 60%, in depressive pseudodementia OPA of 100%.

### Trichotomization of CSF biomarkers

We applied a trichotomization strategy by considering CSF results within 20% of the standard deviation of the mean as borderline. Aβ_1-42_ showed 14.8% of borderline results, 23.3% for pTau and 64.4% for tTau. Regarding combined biomarkers, 11.0% of Aβ_1-42_/Aβ_1-40_, 21.3% of pTau/Aβ_1-42_ and 18.1% of tTau/Aβ_1-42_ were borderline.

When borderline Aβ_1-42_ values were excluded from the analysis, OPA between CSF amyloid biomarkers and amyloid-PET increased by 3% for Aβ_1-42_ and tTau and 2% for pTau. When borderline Aβ_1-42_/Aβ_1-40_ values were excluded, OPA Aβ_1-42_ and amyloid-PET did not change and increased by 2% for pTau and 3% for tTau (Table 2 Supplementary, Table 3 Supplementary).

### Agreement between CSF Aβ_1-42_ with amyloid-PET by CSF analysis tool.

Aβ_1-42_ analyzed with Lumipulse® showed a higher agreement with PET compared to Innotest® (65.4 vs 78.1, *p* = 0.043), as did pTau analyzed with Lumipulse® (65.4 vs 79.4, *p* = 0.027). We did not find differences in tTau (67.9 vs 76.1, *p* = 0.215) or tTau/Aβ_1-42_ (83.3 vs 80.9, *p* = 0.835). Aβ_1-42_/Aβ_1-40_ and Aβ_1-42_/pTau were analyzed only with Lumipulse®.

## Multicenter study

Results reflect the variability corresponding to a 5-center study, emphasizing different sample sizes (from 15 to 94 participants), different CSF biomarkers cutoffs and different laboratory techniques. The greater differences between PPA and NPA were found for Aβ_1-42_. PPA values varied from 51 to 100% between centers, NPA values varied from 49 to 100%, OPA values varied from 61 to 93%, and AUC varied from 0.64 to 0.95. In all cases Aβ_1-42_/Aβ_1-40_, pTau/Aβ_1-42_ and tTau/Aβ_1-42_ improved agreement and AUC compared to single biomarkers (Table 2).

## Discussion

In our study, we analyzed the agreement between CSF biomarkers (Aβ_1-42_, pTau, tTau) and their ratios (Aβ_1-42_/Aβ_1-40_, Aβ_1-42_/pTau, Aβ_1-42_/tTau,) with amyloid-PET in a heterogeneous sample of participants from 5 centers. Different laboratory protocols, CSF analysis techniques (Innotest®, Lumipulse®), biomarker cut-off points and amyloid-PET tracers (18F-Florbetaben, 18F-Flutemetamol) were used. Regarding the diagnoses, there were participants with neurodegenerative diseases, non-neurodegenerative diseases, subjective memory complaints and cognitively unimpaired individuals. Both a LP and an amyloid-PET scan had been performed, in the framework of clinical trials, research projects or a non-conclusive first test. In the five centers, the use of a second CSF biomarker resulted in an increase of the agreement with amyloid-PET. The highest agreement was found in participants with an A + T + N + profile.

Clinical indication of AD CSF biomarkers and amyloid-PET is similar and there are no studies that show if one test is preferable over the other one in clinical practice. It’s well known that lumbar puncture is more invasive, although well tolerated [25], and that amyloid-PET is more expensive. A recent study has explored cost-effectiveness of these two tests in the diagnosis of AD among subjects with early onset cognitive decline, concluding that amyloid-PET is not a cost-effective technique compared to AD CSF biomarkers [26]. Both share the need to establish thresholds for positivity, leading to a binary separation of positive and negative results, while the AD pathophysiological process is more complex, and each biomarker has its own and different trajectory [27]. The LP has the advantage of providing information about the three components of the ATN framework in one test. Other advantages of obtaining CSF through a LP are to allow analyzing other biomarkers such as α-synuclein, 14–3-3 protein or neurofilament light chain, which could be especially useful to complete the diagnostic study if AD CSF biomarkers are negative.

Aβ_1-42_/Aβ_1-40_, pTau/Aβ_1-42_ and tTau/Aβ_1-42_ ratios showed a better agreement with amyloid-PET than individual biomarkers. These results are in line with recent studies [5, 28–30] demonstrating that the use of CSF ratios improves agreement with amyloid-PET over using single biomarkers. Firstly, normalizing Aβ_1-42_ to the concentration of Aβ_1-40_, a peptide that is much more abundant in CSF, may compensate individual differences in amyloid precursor protein processing and provide a more specific information on the pathological amyloidosis deposition [31]. In this line, a recent work demonstrated that the Aβ_1–42_/Aβ_1–40_ ratio in CSF is more strongly associated to tau markers and clinical progression than Aβ_1–42_ alone [32]. In the same way, another recent work showed that global cortex standardized uptake value ratios of amyloid deposition in amyloid-PET correlated highly with CSF Aβ_1-42_/Aβ_1-40_ and moderately with Aβ_1-42_ but not with Aβ_1-40_ [30]. Our results replicate those of Amft et al., that made a comparison between amyloid-PET and CSF biomarkers with pre-defined cut-offs in a clinical cohort with memory deficits, showing that combined biomarkers in CSF, specially pTau/Aβ_1-42_ and Aβ_1-42_/Aβ_1-40_, predicted amyloid-PET result better than Aβ_1-42_ [33]. Secondly, it has been described that CSF Aβ_1-42_ levels can be abnormal earlier in the disease course [34] than amyloid-PET visual read. Therefore, combining Aβ_1-42_ in a ratio with pTau or tTau, markers that are abnormal later in the disease, may correspond better to amyloid-PET visual read.

When comparing ATN profiles, in our study participants with an A + T + N + or A-T-N-profile had the highest agreement between Aβ_1-42_ and amyloid-PET. About 2/3 of the participants with a A-T + N + profile had a positive amyloid-PET, and 50% of the participants with A + T-N- profile had a positive amyloid-PET. In the last group, the positivity of Aβ_1-42_ in CSF but not in the amyloid-PET could be explained by a low concentration of total amyloid peptides (that corrects by normalizing to the concentration of Aβ_1-40_) or a very initial phase of AD, in which Aβ_1-42_ becomes positive in CSF before it does in amyloid-PET.

Several reasons may contribute to the limitation of agreement between both methods in this study: as we mentioned, the clearcut separation between negative and positive patients in relation to a given biomarker is somehow artificial and differs between sites and studies. In our study, when we eliminated the borderline Aβ_1-42_ and Aβ_1-42_/Aβ_1-40_ values from the analysis, we found small changes in the agreement between CSF amyloid biomarkers and amyloid-PET. Therefore, the lack of agreement between the two techniques could not be attributed to the borderline values or could explain only a small part of this lack of agreement. Regarding CSF analysis techniques, automated platforms such as Lumipulse® reduce manual steps as a source of variation; recent studies directly comparing measurements with Lumipulse® with Innotest® showed reduced intra- and inter-assay variability on the Lumipulse® [35]. However, in this study, 34% of CSF biomarkers were determined using Innotest®. In our study, measurements of Aβ_1-42_ and pTau made with Lumipulse ® showed a better agreement with amyloid-PET than those made with Innotest®. However, this is not a study designed to compare these two techniques and there are confounding factors (such as the reason for which the two tests were performed) that are not equally distributed in the two groups. Finally, regarding amyloid-PET analysis, the use of the centiloid quantification scale instead of the visual read may reduce the variability and identify earlier stages of amyloid accumulation [36, 37].

In this multicenter study, each of the participating centers developed their own cut-off points for each biomarker maximizing sensitivity and specificity. Therefore, mean values of biomarkers are different and not comparable between centers.

Time between LP and PET-scan can influence the results of the study. As it is well known, CSF AB1-42 starts to decrease in CSF before amyloid accumulation is detected by PET imaging, and both precede CSF p-Tau and t-Tau increase in CSF. Previous studies described that CSF Aβ_42_ was fully abnormal 5–10 years or more before dementia diagnosis, there was little change in the anatomical extent of amyloid PET over time in individuals with mild AD while it was static by the time the patients became demented. By contrast, both CSF t-tau and p-tau became progressively more abnormal as the time to diagnosis of dementia decreased, in periods of 2.5 years previous to dementia [38, 39]. [Jack, Forster] We set the time limit at 18 months to control that the time difference was not responsible for the lack of agreement between biomarkers. The mean time between both tests was 5 months, a period that would normally elapse in clinical practice between the two tests, and in which we expect no changes or minimal changes in CSF biomarker levels and no changes in the result of the amyloid PET.

This study has some limitations that need to be taken into consideration. In the first place, suspected diagnoses before CSF biomarkers and amyloid PET results were not registered. Therefore, valuable information regarding the agreement of both tests according to initial clinical orientation is lost. Secondly, participants came from different centers attending diverse populations, using different biomarker cut-offs, CSF platform analysis and PET tracers and therefore resembling those of daily clinical practice. But also, some patients participating in clinical assays and research projects may have been carefully selected, with none or few comorbidities, and those with a non-conclusive first tests were probably of high complexity, compared to the characteristics of patients in real-world daily clinical practice. Finally, the number of patients in some of the centers and in some of the diagnostic categories was small.

The main strength of this study is that it is a multicenter study with the participation of 5 centers that used different protocols for CSF biomarker analyses, different cutoffs for biomarkers positivity and different PET tracers, so the results resemble the heterogeneity of daily clinical practice. We are aware of some limitations: diagnoses reported in the study considered the result of biomarkers; since this was a retrospective study, it was not possible to systematically record the clinical diagnosis prior to the biomarkers use. Also, sample size of the 5 centers was different and some of the clinical categories included a small number of participants.

In conclusion, in this heterogeneous multicenter study, combined biomarkers in CSF (Aβ_1-42_/Aβ_1-40_, pTau/Aβ_1-42_, tTau/Aβ_1-42_) were better markers of cerebral amyloid deposition, as identified by amyloid tracers, than single biomarkers (Aβ_1-42_, pTau, tTau). Participants with an A + T + N + profile had a high percentage of positive amyloid-PET scans (96%) and those with an A-T-N- profile of negative amyloid-PET scans (89%), whereas participants with an A + T-N- profile had the same proportion of positive (49%) and negative (51%) amyloid-PET scans.

In conclusion, in this heterogeneous multicenter study, combined biomarkers in CSF (Aβ_1-42_/Aβ_1-40_, ptau/Aβ_1-42_, tTau/Aβ_1-42_) were better markers of cerebral amyloid deposition, as identified by amyloid tracers, than single biomarkers (Aβ_1-42_, pTau, tTau). In participants with A + T + N + and A-T-N- profiles, CSF Aβ1-42 and amyloid-PET were of the same sign, whereas those with a A + T-N- profile had the same proportion of positive and negative amyloid-PET scans.

## Supplementary Information

Below is the link to the electronic supplementary material.

Supplementary file1 (DOCX 23 KB)

## Data Availability

The data that support the findings of this study are not openly available due to reasons of sensitivity and are available from the corresponding author upon reasonable request.
