# Supplementary material for: Agreement of cerebrospinal fluid biomarkers and amyloid-PET in a multicenter study
Source: Eur Arch Psychiatry Clin Neurosci. 2023 Oct 28;275(1):257–66. doi: 10.1007/s00406-023-01701-y (PMC11799063; doi:10.1007/s00406-023-01701-y)
Supplement: Supplementary file 1 — Supplementary file1 (DOCX 23 KB) [file 406_2023_1701_MOESM1_ESM.docx]

**SUPPLEMENTARY MATERIAL**

**Supplementary Table 1**. Demographical and clinical data according to ATN profile.

| Biomarker AT(N) profile | A+T+N+ | A-T-N- | A+T-/N- | A-T+/N+ |
| --- | --- | --- | --- | --- |
| Total  PET+/PET-  Age  Sex female/male (% fem)  MMSE (n=222)  APOEε4 +/- (%+) (n=212)  Follow up diagnosis  MCI due to AD  Dementia due to AD  Dementia with Lewy bodies  Vascular dementia  Cognitively unimpaired  Frontotemporal dementia  MCI non degenerative  SMC   MCI degenerative non-AD  Depressive pseudodementia | 77  74/3 (96%)  69.6 (8.3)  40/37(52%)  23.5 (4.5)  38/35 (52%)  37 (48%)  21 (27%)  17 (22%)  1 (1%)  1 (1%)  -  -  -  -  - | 46  5/41 (11%)  67.4 (8.8)  27/19 (59%)  25.9 (2.8)  8/33(20%)  1 (2%)  3 (7%)  15 (33%)  2 (4%)  -  7 (15%)  13 (28%)  4 (9%)  -  1 (2%) | 74  40/34 (54%)  66.2 (10.4)  37/37(50%)  25.0 (4.6)  30/35(46%)  19 (26%)  13 (18%)  19 (26%)  1 (1%)  4 (5%)  10 (14%)  7 (9%)  1 (1%)  -  - | 39  20/19 (51%)  68.3 (8.2)  28/11 (72%)  24.2 (4.6)  12/22 (35%)    9 (23%)  12 (31%)  2 (5%)  -  -  6 (15%)  2 (5%)  2 (5%)  6 (15%)  - |

PET, positron emission tomography; MMSE, Mini-mental state examination; MCI, mild cognitive impairment; AD, Alzheimer’s disease, CSF, cerebrospinal fluid; LP, lumbar puncture; SMC, subjective memory complaints.

**Supplementary Table 2.** Effect of the removal of Aβ_1-42_ and Aβ _1-42_/Aβ_1-40_ borderline cases in overall percent agreement (OPA) between CSF amyloid biomarkers and amyloid-PET.

| CSF amyloid biomarker | OPA  N=236 | OPA  Discarding borderline Aβ_1-42_  N=201 | OPA  Discarding borderline Aβ_1-42_/Aβ_1-40_  N=138 |
| --- | --- | --- | --- |
| Aβ_1-42_ | 74% | 77% | 74% |
| pTau | 75% | 77% | 77% |
| tTau | 73% | 76% | 76% |
| Aβ_1-42_/Aβ_1-40_ | 86% | 87% | 88% |
| pTau/Aβ_1-42_ | 88% | 88% | 88% |
| tTau/Aβ_1-42_ | 82% | 86% | 83% |

Aβ_1-42_, amyloid- βeta_1-42_; Aβ_1-40_, amyloid- βeta_1-40_ CSF, cerebrospinal fluid; OPA, overall percent agreement; PET, positron emission tomography; pTau, phosphorilated tau; tTau, total tau

**Supplementary Table 3.** Agreement between CSF Aβ_1-42_ or Aβ _1-42_/Aβ_1-40_ and amyloid-PET. Comparison of dichotomization and trichotomization strategies.

| ATN status (AB_1-42_)  Dichotomic (+/-) | All participants | Amyloid-PET Result  (+/-) | ATN status (AB_1-42_)  Trichotomic (+/-/borderline) | All participants | Amyloid-PET Result |
| --- | --- | --- | --- | --- | --- |
| A+T+N+ | 77 | 74+/3- | A+T+N+ | 72 | 69+/3- |
| A+T+N- | 8 | 5+/3- | A+T+N- | 7 | 4+/3- |
| A+T-N+ | 5 | 3+/2- | A+T-N+ | 4 | 3+/1- |
| A+T-N- | 61 | 32+/29- | A+T-N- | 51 | 27+/24- |
| A-T-N- | 46 | 5+/41- | A-T-N- | 36 | 2+/34- |
| A-T+N+ | 31 | 20+/11- | A-T+N+ | 24 | 13+/11- |
| A-T-N+ | 5 | 0+/5- | A-T-N+ | 4 | 0+/4- |
|  |  |  | A_borderline_T+N+ | 12 | 12+/0- |
|  |  |  | A_borderline_T+N- | 1 | 1+/0- |
|  |  |  | A_borderline_T-N+ | 2 | 0+/2- |
|  |  |  | A_borderline_T-N- | 20 | 8+/12- |
| ATN status (AB_1-42_/AB_1-40_)  Dichotomic (+/-) | All participants | Amyloid-PET Result  (+/-) | ATN status (AB_1-42_/AB_1-40_)  Trichotomic (+/-/borderline) | All participants | Amyloid-PET Result |
| A+T+N+ | 68 | 64+/4- | A+T+N+ | 67 | 63+/4- |
| A+T+N- | 8 | 5+/3- | A+T+N- | 7 | 5+/2- |
| A+T-N+ | 2 | 1+/1- | A+T-N+ | 1 | 1+/0- |
| A+T-N- | 23 | 16+/7- | A+T-N- | 15 | 10+/5- |
| A-T-N- | 45 | 5+/40- | A-T-N- | 40 | 3+/37- |
| A-T+N+ | 4 | 2+/2- | A-T+N+ | 4 | 2+/2- |
| A-T-N+ | 4 | 0+/4- | A-T-N+ | 3 | 0+/3- |
|  |  |  | A_borderline_T+N+ | 1 | 1+/0- |
|  |  |  | A_borderline_T+N- | 1 | 0+/1- |
|  |  |  | A_borderline_T-N+ | 2 | 0+/2- |
|  |  |  | A_borderline_T-N- | 13 | 8+/5- |

Aβ_1-42_, amyloid- βeta_1-42_; Aβ_1-40_, amyloid- βeta_1-40_ CSF, cerebrospinal fluid; PET, positron emission tomography.
